# Implementation and evaluation of a primary diabetes prevention programme for young adult employees in Japan: A non‐randomized controlled trial

**DOI:** 10.1002/nop2.372

**Published:** 2019-09-26

**Authors:** Hitomi Nagamine, Xiaowei Lyu, Kayo Maruyama, Kumiko Morita

**Affiliations:** ^1^ Community Health Promotion Nursing Graduate School of Health Care Sciences Tokyo Medical and Dental University Tokyo Japan

**Keywords:** diabetes, employee, health education, occupational health, primary prevention, young adults

## Abstract

**Aim:**

To determine the effects of a primary diabetes prevention programme created for healthy young adults.

**Design:**

This study was a non‐randomized controlled trial.

**Methods:**

The participants were 20–39‐year‐old employees of two automobile sales companies. The intervention group (*N* = 154) received six original educational brochures and films created specifically for young adults, while the control group (*N* = 157) received none. Data were collected pre‐intervention and immediately after and 10 weeks after intervention. Change in knowledge about diabetes, its prevention and health management were measured.

**Results:**

Overall, 129 interventions and 141 controls completed the trial. In items related to diabetes prevention, the intervention group increased their knowledge relative to controls (all *p* < .05). Awareness of susceptibility to diabetes also increased more in the interventions (*p* = .029). The interventions also improved more with items related to dietary behaviour (*p* < .05). This trial has been registered with UMIN‐CTR clinical trial (UMIN000023749).

## 1 INTRODUCTION

In Japan, the number of patients living with a diagnosis of type II diabetes mellitus (T2DM) was estimated to be 10 million (The Ministry of Health, Labor, & Welfare, 2016b). There have been several attempts to prevent onset and progression of T2DM for high‐risk populations (The Ministry of Health, Labor, & Welfare, 2016a). Irregular lifestyles or the overweight that accompanies them can cause T2DM. According to a national survey, individuals aged 20–39 years were found to have unhealthy lifestyle habits more than other generations (The Ministry of Health, Labor, & Welfare, 2016b). A different report found that individuals in their late twenties and early thirties increased their body mass index (BMI) rapidly (Umakoshi, Sumino, & Kitai, 2001). Furthermore, Wainai (2003) found that a high proportion of individuals experienced weight gain immediately after landing their first job. Yet another Japanese study found that the presence of obesity in their forties increased the risk for onset of T2DM significantly (Seki et al., 2002). Weight reduction is difficult for obese individuals and if attempts to lose weight do not result in tangible results, individuals are unlikely to maintain healthy lifestyles; behaviour modification stagnates or even regresses (Tominaga, Takigawa, & Sakane, 2010). These previous studies suggest that unchecked inappropriate lifestyles in young adults may be increasing obesity rates and the prospective risk of T2DM. Three decades of cohort studies have indicated that Japanese are at high risk of lifestyle‐related diseases including T2DM even if their BMI is only moderately high (Yoshiike et al., 2000). In other words, Japanese are predisposed to lifestyle‐related diseases in presence of modest obesity. This may be related to a finding of a previous study that insulin secretory capacity of East Asians is much lower than that of Caucasians (Yabe, Seino, Fukushima, & Seino, 2015). If we want to prevent T2DM, such results suggest that young adults should be provided with guidance in achieving and maintaining a favourable lifestyle and education about their health status.

### 1.1 Background

The Japanese Ministry of Health, Labor and Welfare (The Ministry of Health, Labor And Welfare, 2007) have deemed it important to prevent lifestyle‐related diseases in individuals aged less than 40 through education relating to appropriate lifestyle habits. Recently, some municipal governments in Japan have launched health check‐ups for adults aged 39 or less, with the hopes of detecting lifestyle‐related diseases early (Soga, Shirai, & Ijichi, 2013). Cost‐effectiveness of screening for diabetes at the age of 35 has been confirmed in the United States (Chung, Azar, Baek, Lauderdale, & Palaniappan, 2014). Thus, the importance of early identification of individuals with diabetes risk and getting them preventive education is gradually being recognized. However, we know of no studies which have looked at efficacy of primary diabetes prevention programmes intended for healthy young adults.

Nagamine, Yamamoto, and Morita (2018) conducted a preliminary study of diabetes prevention among young adults. The results of that study showed that having a habit of self‐health monitoring such as measuring body weight daily and having a high interest in methods for maintaining health positively affected the amount of knowledge they had about diabetes. Moreover, that study confirmed that young adults were highly interested in receiving health guidance and that increased awareness affected their willingness to participate in health guidance programmes (Nagamine et al., 2018).

### 1.2 Aim

The aim of this study was to devise a primary diabetes prevention programme for young adults based on the results of our preliminary study (Nagamine et al., 2018) and to examine the effects of this programme. In this study, we examined whether: our programme: (a) enhanced knowledge, cognition related to their health management and diabetes prevention for young adults; and (b) improved their lifestyle behaviour.

## 2 METHOD

### 2.1 Definition of terms

Young adults: adults aged 20–39 of both sexes.

### 2.2 Study design

This study was a non‐randomized controlled trial for evaluating the effects of primary preventive intervention targeting young adults. All participants were allocated to either the intervention or control arm.

### 2.3 Participants

The participants of this study were employees who worked as automobile mechanics or office clerks at two automobile sales companies in Japan. The inclusion criterion was the following: employees of either sex, aged 20–39. The exclusion criterion was the following: participants who had previously received a health education programme about lifestyle modification from medical personnel. Questionnaires for self‐administration were distributed to all employees aged 20–39 (*N* = 311) along with an explanatory pamphlet where we asked recipients interested in taking part in the study to fill out and return the questionnaire and those not interested to discard the questionnaire. We asked those meeting the exclusion criteria not to participate in this study. These companies have a 6‐day work week, with no night shift. The two companies had 19 and 44 plants each. The number of employees at each plant was small (1–9 persons) and varied considerably. We randomly assigned participants at each plant so the intervention and control group were roughly equal in size. As a result, 154 participants were assigned to the intervention group and 157 participants to the control group. Participants were blinded to their allocation status.

The minimum sample size was estimated using G‐power 3.1 (Faul, Erdfelder, Lang, & Buchner, 2007). We planned to analyse the differences between the intervention and the control group using paired data. The estimated number was 220 (110 per group) with an effect size of .5, an *α* of .05 and a power of .95.

### 2.4 Development of the primary diabetes prevention programme for young adults

#### 2.4.1 Media

Our intervention programme consisted of six brochures, six films and a tape measure for measuring girth. It was a population based rather than individualized approach, because the preliminary survey had found that the health risk level of the study participants was low (Nagamine et al., 2018). The material covered in the brochures and films was identical, and participants could choose whichever of the two media they preferred. We also prepared the programme so that participants could access the films via their smartphones. The programme contents were produced by a nurse experienced in health consultations for patients with diabetes and were thoroughly vetted by healthcare professionals. Brochure details are described in Table 1. The six brochures were distributed every 2 weeks, two at a time for a total of three times. The films were created using Camtasia studio^®^ (TechSmith corporation) and Voice Sommelier Neo^®^ (HITACHI solutions create corporation). The control group received only the tape measure during the intervention period. We gave all control group participants the six brochures and films after the end of the study.

**Table 1:** The contents of the primary diabetes prevention programme

1. Weight control and practical use of annual health check‐ups (7pages)		Element
Feedback about results of previous survey	General health status of study participants (e.g., the disease rate and the rate of obesity)	Ⅰ
Measurement of body weight (BW)	Explain how to include the habit of observing body weight in daily life	Ⅳ
Calculate and assess present BMI	Show how to enter body weight in a worksheet and calculate BMI	Ⅱ
Calculate appropriate BW	It is possible to calculate difference between present and optimal BW, and provide valid targets for weight reduction	Ⅱ
Specific targets for weight control to achieve normal weight	Explain how energy intake has to be reduced 240 kcal/day to reduce weight by 1 kg/month, that is equivalent to a bowl of rice (about 150 g)	Ⅳ
Metabolic syndrome (Mets)	Diagnostic criteria and how to measure waist circumference	Ⅱ, Ⅳ
Reasonable and safe ways of setting goals for weight reduction	Explain that 5% weight reduction in Mets improves blood pressure, blood glucose and serum lipid levels	Ⅱ, Ⅳ
How to assess the results of an annual health check‐up	Explain what is normal with respect to FBS, HbA1c, lipid metabolism and kidney function. Define the values at which lifestyle modification or early medical examination is recommended	Ⅳ
Provide information about government sponsored health check‐ups for young adults	Introduce locally offered inexpensive health check‐ups	Ⅳ
2. Explain about Diabetes (6pages)		Element
Feedback about results of previous survey	The survey suggested that young adults who knew more about diabetes retained high health management consciousness, and had better lifestyle habits	Ⅰ
Financial burden of diabetes	Inform that early lifestyle modification also has financial benefits	Ⅳ
Carbohydrate metabolism and the function of insulin	Illustrate the mechanism of carbohydrate metabolism and explain that lack of exercise and obesity may exacerbate insulin resistance	Ⅲ
Pathology of type 2 diabetes (T2DM)	Explain the difference between type 1 and 2 diabetes and grades of diabetes with illustrations	Ⅲ
Genetic vulnerability to T2DM	Explain that diabetes prevention is possible with lifestyle modification in spite of genetic Japanese vulnerability to T2DM	Ⅲ
Symptoms which suggest presence of diabetes	Characteristic somatic symptoms of diabetes. How to judge when to get a medical examination	Ⅱ, Ⅲ
Diabetic complications	Explain main complications with illustrations	Ⅲ
3. Well‐balanced diet (1) (6pages)		Element
Feedback about results of previous survey	Summarize eating habits uncovered in prior survey	Ⅰ
Examples of a well‐balanced diet	Illustrate optimal meal portions and content. An optimal meal contains a staple, a main dish and a side dish. Photographs and units of measurement (g, ml) provided	Ⅳ
Diurnal fluctuations of blood sugar	Explain that irregular eating habits damage blood vessels and cause diabetes	Ⅱ
Optimal caloric consumption	Optimal energy intake per meal, and adequate quantity of a dish (a staple food, a main dish and a side dish), using photographs and words such as “a palm size” to help with visualization	Ⅳ
Optimal vegetable organic consumption	Vegetable consumption should be about 350 g per day or a quantity about the “size of two palm fulls" per meal. Explain good effects of vegetables and eating vegetables at the start of a meal. Introduce how to cook steamed vegetables, to utilize a bagging cut vegetables which is possible to buy at convenience stores in Japan and inexpensive vegetables	Ⅳ
How to make good meal choices at convenience stores and restaurants	Present how to select a meal wherein nutrition balance is considered with photographs	Ⅳ
4. Well‐balanced diet (2) (6pages)	Element	Element
Guideline for salt intake	Provide a worksheet that enables salt intake calculation	Ⅱ
Concrete examples of methods for cutting down on salt	Explain the proper way to use soy sauce or spices for reduce salt consumption. Draw attention to foods with high salt content such as proceed foods (e.g. sausages), miso soup, and Japanese pickles. Explain how to read nutrition content labels	Ⅳ
List of foods that contain a lot of salt	Explain the amount of salt contained in typical servings of noodle soups, fast foods, donburimono (rice with topping) or the dishes served at izakaya (Japanese‐style bar)	Ⅳ
About Fruits and snacks	Illustrate the appropriate amount of fruits and the allowed amount of snacks per day	Ⅳ
The amount of sugar contained in beverages	Illustrate the amount of sugar within each beverage using an image of sugar sticks	Ⅳ
5. Exercise (8pages)		Element
Feedback about results of previous survey	Show the number of those who want to exercise daily and kinds of exercise in which this study's participants have interest	Ⅰ
Effects of physical activity	Explain the difference between “exercise ”and “living activity”	Ⅳ
	Show good effects of physical activity that is evidence‐based	
Physical activity goals	Recommended amount of physical activity per week for health promotion; show the amount of activity necessary for specific activities given by study participants in survey (e.g. it need to take 40 min for swimming by slow speed)	Ⅳ
Concrete examples of exercise	Propose concrete examples of physical exercise in consideration of characteristics specific to their occupation	Ⅳ
	Introduce radio gymnastics and easy methods for muscle training	
Checklist for exercising safely	Show the way to check own state of health before starting exercise	Ⅱ
Methods for improving knee and back pain	Illustrate effective stretching exercise methods with some photographs	Ⅳ
Provide information about the “Health Point System”	The Health Point System aims to promote health. It is implemented by local governments for citizens, and rewards users with cash for exercising or performing health promotion activities	Ⅳ
6. Smoking and alcohol intake (6pages)		Element
Feedback about results of previous survey	Show previous survey results related to smoking and alcohol intake among the study participants, and compare them with national averages	Ⅰ
Smoking	Introduce merits of smoking cessation (such as reducing risk of disease onset, economic merits), appropriate methods of smoking cessation by using self‐check tool (the nicotine dependency determination tool)	Ⅳ
Alcohol intake	Appropriate limits of alcohol intake/day, relation to lifestyle diseases and the importance of having more than two alcohol‐free days/ week	Ⅱ, Ⅳ

Note

This table shows a summary of the contents of the various sections of the educational programme.

#### 2.4.2 Construction of the education programme using conceptual frameworks

The programme contents were based on two conceptual frameworks, the Health Belief model (HBM) and the Revised Health Promotion model (Revised HPM). The HBM describes two conditions under which individuals take action: one, the individual is convinced that the benefit of taking action to protect health overweighs the barriers and two, the individual perceives threat of a disease (Pender, 2014). The HBM model attaches importance to “fear” and “threat” as motivators for healthy behaviour (Pender, 2014). A criticism of the HBM is that healthy individuals would be less motivated to make an effort to prevent a threat in the distant future (Pender, 1997, 2014). Because our programme targeted healthy individuals, we also incorporated the Revised HPM, an approach‐oriented model that has a different view on how individuals are motivated to pursue healthy behaviours (Pender, 2014). The Revised HPM is predicated on the following: Individuals place value on growth that they consider positive and they try to balance change and stability. Individuals have potential for self‐directed change in their health behaviour, and they are also able to change in a positive direction under the influence of environmental factors that include guidance from healthcare professionals (Pender, 1997, 2014).

By combining the frameworks of the HBM and revised HPM, we attempted to get healthy individuals to recognize their future risk for diabetes and to select and practice voluntarily, health behaviour within their means from among several recommended.

1. Giving feedback for prior related behaviour (Element l)

According to the Revised HPM, prior related behaviour indirectly influences health‐promoting behaviours and feedback about past behaviours leads to self‐efficacy (Pender, 2014). The term self‐efficacy refers to confidence in one's self and ability to effect change. In the preliminary study we referred to above, we had surveyed the same study population about their lifestyle behaviour (Nagamine et al., 2018). Therefore, for each of the brochures, the first informational content was feedback about the results of the prior survey (Table 1).

- Assessment of regular health check‐up results (Element ll)

The Revised HPM proposes that awareness of factors such as BMI or blood test results directly influence behaviour‐specific cognition and affect health promotion behaviour (Pender, 1997). Therefore, we educated participants about appropriate body weight, caloric goals and normal values of blood tests. Furthermore, we provided instruction on how deviations from normal progress and what to be on the alert for, even if current test data are all within normal limits.

- Perceived susceptibility for and severity of diabetes (Element lll)

This element was based on the HBM. Japanese have a genetic vulnerability to T2DM (Yabe et al., 2015). A previous study found that education targeting youth about the susceptibility for and severity of T2DM prompted modification of exercise habits (Satoh, Mizuki, Kurosawa, & Nishizawa, 2012). We therefore added information about susceptibility to diabetes and the seriousness of diabetic complications.

- Maximizing perceived benefits of action and minimizing perceived barriers (Element lV)

Several experimental studies have confirmed that the perceived benefits and barriers for action could be important predictors of health behaviours (Pender, 1997). Perceived benefits of action are expectation of positive consequences that can be obtained by engaging in a specific health behaviour (Pender, 2014). Benefits may be intrinsic (e.g. reduced fatigue) or extrinsic (e.g. monetary rewards). While intrinsic benefits can be powerful motivators for sustaining healthy behaviours, extrinsic benefits may be highly significant for getting certain behaviours going. Our education programme contained information that largely emphasized extrinsic benefits. Secondly, perceived barriers are considered to be more powerful than perceived benefits as predictors for a particular health behaviour (Pender, 1997). Barriers usually consist of negative perceptions related to a particular behaviour such as expense, time‐burden and difficulty. Because this educational programme targeted healthy participants, dietary restrictions and exercise regimens were not onerous. We made an effort to minimize the barrier effect by emphasizing content that could be easily incorporated into daily routines. For example, we provided information about easy cooking methods for vegetables, inexpensive vegetables and how to select a healthy meal at convenience stores or restaurants. We also provided information about health check‐ups offered by local governments which young adults can use at low cost and a system called the "Health Point System" (Table 1).

#### 2.4.3 Applying knowledge obtained from the previous survey

The preliminary survey conducted to design this education programme suggested that educating young adults about health management including checking their own health condition or health check‐ups regularly increased their awareness and knowledge about health management (Nagamine et al., 2018). Therefore, we included education about how to assess one's health and results of medical check‐ups and exams. This preliminary survey also suggested that those who were interested in modifying their exercise and eating habits were more probably to show an interest in receiving health guidance. Accordingly, we made an effort to incorporate practical and easily understandable instructions into the education programme.

### 2.5 Data collection

To maintain anonymity of participants, we distributed the multiple questionnaires inside a folder that was marked with a coded ID for each participant that was kept at the workplace. At designated times, we sent all participants a prompt to retrieve the appropriate questionnaire from their folder, respond and return to us. Baseline data were collected in November 2016. Distribution of educational media to the intervention group was implemented over the 6 week period extending from January–February 2017. For the intervention group, questionnaires were collected twice, at the end of intervention and 4 weeks later (at the ends of February and March 2017), whereas data collection from the control group was only performed one time (at the end of March 2017).

### 2.6 Measurements

Demographic variables included age, gender, marital status, existence of family members living in the same house, educational level, occupational category and health status (whether obesity or disease caused by obesity had ever been pointed out by health professionals). These were queried in the questionnaire at baseline.

Outcome measurements were developed and tailored to measure the change in participant's knowledge, cognition and behaviour related to their health management and diabetes prevention.

In order to measure the change in knowledge, we asked them to answer questions about health management and diabetes prevention (knowledge items). The participants selected their responses from a 5‐point ordinal scale from 1 (disagree)–5 (agree). The details of these items are shown in Table 3. To measure the change in cognition of managing their health condition, we used the modified Perceived Health Competence Scale (PHCS) (Togari, Yamazaki, Koide, & Miyata, 2006b). The PHCS measures the extent to which an individual feels capable of modifying health‐related lifestyle and behaviour. This scale consists of eight questions and uses a 5‐point Likert scale response option. The theoretical score range was between 8–40 with higher scores indicating greater levels of reported perceived competence for managing one's health. Cronbach's coefficient *α* of the PHCS was .869 in one study (Togari et al., 2006b). Furthermore, to measure the extent to which an individual is aware of the susceptibility for diabetes, we used a Visual Analogue Scale (VAS). The question asked was as follows: “To what extent do you think of diabetes as a problem relevant to you?” The score range was between 0–100 mm with higher scores indicating greater levels of perception of diabetes as familiar and more important for an individual.

About change in behaviours, we studied the following items: dietary behaviours, amount of physical activity, the stage of behaviour change and waist circumference size.

Questions about dietary behaviours were constructed based on the Health Assessment Manual (Health Assesment Kentou Iinkai, 2000) and the document Health Information about Lifestyle‐related Disease Prevention (The Ministry of Health, Labor, & Welfare, 2008). The questions are listed in Table 3. The participants selected their responses from a 5‐point scale: 1 (no); 2 (not much); 3 (not sure); 4 (somewhat); and 5 (yes). For evaluating self‐reported recent average level of physical activity, we used the variable METS minutes per day from the International Physical Activity Questionnaire Short Version (Craig et al., 2003; Murase, Katsumura, Ueda, Inoue, & Shimomitsu, 2002).

About the stage of behaviour changes, based on the Transtheoretical model (Pender, 2014; Prochaska & Velicer, 1997), we asked participants to evaluate their present stage in five‐stages for the following items: dietary habit, exercise habit and smoking, the last of which was only asked of participants who smoked regularly. About the change in waist circumference, we asked participants to measure their waist circumference using a tape measure which was distributed by researchers in advance.

In the intervention group, the changes in items related to knowledge and cognition were measured at 6 weeks and the items related to behaviour changes (diet behaviour, amount of physical activity) were asked at 10 weeks (4 weeks after this study intervention was completed). Moreover, about the stage of behaviour changes and the waist circumference, we asked the intervention group twice, at 6 and 10 weeks. In the control group, all of the items were asked at 10 weeks.

### 2.7 Data analysis

Statistical analysis was performed using IBM SPSS Statistics ver. 22 for Windows and a *p*‐value of <.05 was considered statistically significant. For comparing the differences in characteristics between two groups, we employed Fisher's exact test and the Mann–Whitney *U* test. To analyse changes in outcome variables in each group, the Wilcoxon signed‐rank test was implemented. For comparing the differences in outcome between the intervention and control groups, we used the Mann–Whitney *U* test.

## 3 RESULTS

**Figure 1:**
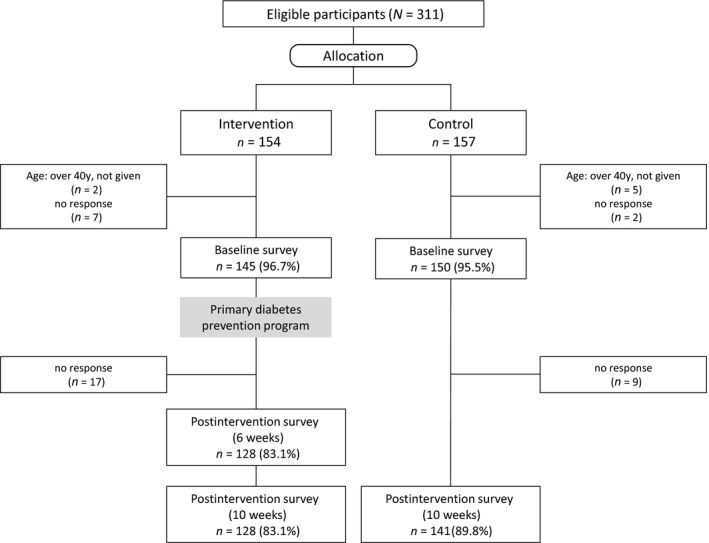
Trial flow chart of eligible participants in the intervention group and the control group

The study participation flow chart is shown in Figure 1. Of 311 eligible participants, 154 and 157 were allocated to the intervention and control groups, respectively. Of these, resulting in a total of 128 intervention group participants (83.1%) and 141 control group participants (89.8%) in our study.

The characteristics of each group at baseline are shown in Table 2. As expected, no demographic or health status factors were found to be different between the two groups. Table 3 shows the effects of the education programme. The intervention group demonstrated a significant improvement in nine of 12 items related to knowledge related to diabetes prevention (*p* < .001 for all). With respect to PHCS scores, a miniscule though statistically significant decrease was observed in both intervention and control groups (both, *p* < .05), but there was no difference in the score change between the two groups after intervention (*p* = .571). Visual Analogue Scale, a measure of awareness about susceptibility to diabetes, significantly increased in the intervention group (*p* < .001). With regard to dietary behaviours, there were nine items. Only two of the nine showed improvements after the education programme. They were as follows: “I take more than 20 min per meal (*p* = .025),” and “I consume the necessary amount of vegetable per day (*p* = .014).” Amount of physical activity did not increase with education, mean 936 (*SD* 1,270) METs before, 722.25 (*SD* 1,053) after (*p* = .51).

**Table 2:** The attribution of this study participants

	Intervention (*N* = 128)		Control (*N* = 141)		*p*
	Mean ± *SD*, *N* (%)		Mean ± *SD*, *N* (%)		
(1) Age (year)^a^	28.7 ± 5.6		28.4 ± 5.3		.682
(2) Sex, male^b^		114 (89.1)		123 (87.2)	.708
(3) Marital status, unmarried^b^		88 (68.8)		93 (66.4)	.697
(4) Family living together^b^		116 (90.6)		134 (95.0)	.233
(5) Educational attainment^b^, ^c^					
Schools or departments specialized in automobile maintenance technics		115 (89.8)		122 (86.5)	.454
Collages (other than the above)		1 (0.8)		2 (1.4)	
Junior university (other than the above)		3 (2.3)		4 (2.8)	
University (other than the above)		9 (7.0)		12 (8.5)	
Other		0 (0.0)		1 (0.7)	
(6) Occupation^b^, ^d^					
Automobile mechanics		95 (74.2)		111 (78.7)	.385
Service staff		29 (22.7)		27 (19.1)	
Head of the division (plant manager)		4 (3.1)		2 (1.4)	
No answer		0 (0.0)		1 (0.7)	
(7) Health status (allow multiple answers^b^)					
Obesity		12 (9.4)		17 (12.1)	.557
Metabolic syndrome		5 (3.9)		7 (5.0)	.773
Hypertension		5 (3.9)		1 (0.7)	.106
Hyperlipidemia		2 (1.6)		0 (0.0)	.225
Diabetes/pre‐diabetes		2 (1.6)		2 (1.4)	1.000
Heart disease		1 (0.8)		1 (0.7)	1.000
Other		13 (10.2)		6 (4.3)	.093
(8) Waist circumference (cm)^a^					
Man	*N* = 112	81.7 ± 9.4	*N* = 121	81.5 ± 9.3	.884
Women	*N* = 14	71.5 ± 5.6	*N* = 18	72.7 ± 9.8	.722

a Mann–Whitney *U* test.

b Fisher's exact test.

c Participants were divided into the following two groups: people who had graduated from schools or departments specializing in automobile maintenance or not, and compared the difference between these two groups.

d Participants were divided into following two groups: automobile mechanics or not, and compared the difference between these groups.

**Table 3:** Changes in parameters from baseline to education programme between two groups

Variable	Intervention (*N* = 128)							Comparison of within groups	Control (*N* = 141)							Comparison of within groups	Comparison of between groups
	*N*	Baseline			Postintervention			*p* a	*N*	Baseline			Postintervention			*p* a	*p* b
		Median	Mean	*SD*	Median	Mean	*SD*			Median	Mean	*SD*	Median	Mean	*SD*		
Knowledge about diabetes prevention^c^																	
I know about target weight value and the methods of weight control	128	3.0	2.70	1.22	3.0	2.88	0.98	.145	141	3.0	2.84	1.12	3.0	2.70	1.06	.114	.029
I know how to assess the results of an annual health check‐up	128	3.0	2.77	1.19	3.0	2.88	1.02	.278	141	3.0	3.14	1.08	3.0	2.79	1.03	.001	.002
I am knowledgeable about diabetes	128	3.0	2.91	1.29	3.0	3.13	1.04	.063	141	3.0	3.06	1.15	3.0	2.96	1.23	.387	.035
I know what kind of situations I should have a medical examination on the suspicion of diabetes	128	2.0	2.19	1.16	3.0	2.71	1.08	.000	141	2.0	2.37	1.08	2.0	2.35	1.11	.750	.000
I know what blood sugar should be controlled at to prevent progression of diabetes	128	1.0	1.93	1.21	3.0	2.41	1.11	.000	140	2.0	1.99	1.07	2.0	1.81	0.89	.122	.000
I know what a well‐balanced diet looks like to prevent lifestyle diseases	128	2.0	2.38	1.17	3.0	2.78	1.12	.002	141	3.0	2.77	1.06	2.0	2.37	1.06	.000	.000
I know the appropriate number of calories I should take per day	128	2.0	2.21	1.19	3.0	2.72	1.13	.000	141	2.0	2.50	1.08	2.0	2.21	1.08	.005	.000
I know the recommended daily amount of vegetable intake	128	2.0	2.09	1.13	3.0	2.70	1.13	.000	141	2.0	2.42	1.04	2.0	2.28	1.12	.148	.000
I know the recommended daily amount of fruit intake	128	2.0	1.97	1.06	3.0	2.58	1.13	.000	141	2.0	2.36	1.04	2.0	2.18	1.08	.108	.000
I know the daily allowed amount of snacks	128	2.0	1.89	0.99	2.5	2.45	1.02	.000	141	2.0	2.32	1.04	2.0	2.17	1.06	.114	.000
I know the necessary amount of daily physical activity to prevent lifestyle disease	128	2.0	1.96	1.00	3.0	2.55	1.08	.000	141	2.0	2.33	1.04	2.0	2.11	0.99	.017	.000
I know the appropriate amount of daily amount of alcohol to prevent lifestyle disease	128	2.0	1.94	1.02	3.0	2.52	1.08	.000	141	2.0	2.35	1.04	2.0	2.16	1.06	.073	.000
The modified perceived health competence scale (PHCS)	126	24.0	23.27	5.94	24.0	22.47	5.03	.030	137	23.0	23.63	5.74	23.0	22.50	5.82	.004	.571
Cognition for the susceptibility for diabetes (VAS, mm)	128	49.0	46.53	31.10	54.0	55.16	27.67	.000	139	51.0	51.18	28.70	52.0	53.21	26.77	.433	.029
Eating behaviour^c^																	
I take more that 20 min per meal	128	2.0	2.41	1.41	3.0	2.65	1.33	.025	141	3.0	2.71	1.41	2.0	2.50	1.30	.081	.017
I have a meal at regular time	128	2.0	2.14	1.28	2.0	2.07	1.10	.681	141	2.0	2.25	1.15	2.0	2.11	1.14	.176	.597
I sometimes skip meals (r)^d^	128	2.0	2.75	1.57	3.0	2.73	1.56	.880	141	2.0	2.67	1.51	2.0	2.71	1.57	.699	.806
I have a well‐balanced meal with a staple, a main dish and a side dish	128	2.0	2.40	1.11	3.0	2.56	1.06	.087	141	3.0	2.64	1.08	3.0	2.57	1.08	.491	.037
I make meal choices considering the appropriate amount of energy intake per day	128	2.0	2.20	1.21	2.0	2.27	1.05	.473	141	2.0	2.21	0.99	2.0	2.19	1.06	.891	.321
I consume the necessary amount of vegetable per day (350 g)	128	2.0	1.82	1.04	2.0	2.05	0.93	.014	141	2.0	1.90	1.01	2.0	1.94	0.94	.614	.056
I am careful of too much salt intake	132	2.0	2.38	1.12	3.0	2.52	1.09	.231	141	3.0	2.60	1.16	3.0	2.46	1.05	.098	.067
I consume fruits every day while being careful of not overeating	132	2.0	1.99	1.07	2.0	2.15	1.04	.147	141	2.0	2.13	1.06	2.0	2.13	1.01	.934	.371
I am careful not to consume too many snacks or soft drinks	132	3.0	2.45	1.20	3.0	2.59	1.15	.181	141	3.0	2.63	1.19	3.0	2.55	1.13	.420	.222
Exercise																	
The amount of physical activities (METS minutes per day)	120	228.4	935.72	1,270.32	162.4	722.25	1,053.01	.501	127	222.0	911.84	1,410.05	265.7	984.96	1,455.38	.119	.097

a Wilcoxon signed‐rank test.

b Mann–Whitney *U* test.

c 5‐point Likert scale, 1 = no, 2 = not much, 3 = not sure, 4 = somewhat, 5 = yes.

d (r) means the scale was reversed with 1 being yes and so forth.

Table 3 also gives comparisons of the intervention group versus the control group. The difference in parameter values before and after education in the intervention group was compared with difference in parameter values before and after a 10‐week period in the control group. The control group essentially had to respond to the same survey twice with a 10‐week period in between with no health education.

Differences in magnitude of improvements between the two groups largely paralleled the intervention results in that the education programme significantly improved the intervention group parameter values, while not surprisingly, the control group values varied little over 10 weeks. Compared with the controls, the intervention group improved their VAS score (*p* = .029) and dietary behaviour items: “I take more than 20 min per meal (*p* = .017),” and “I have a well‐balanced meal with a staple, a main dish and a side dish (*p* = .037).” Interestingly, though not necessarily statistically significant, control group value means worsened over 10 weeks for most parameters with a few exceptions, suggesting that the mere taking of the questionnaire had made some participants feel less secure about their knowledge and health habits.

Table 4 shows change to waist circumference, which was analysed by gender. In the intervention group, there were no significant changes in either gender after 6 and 10 weeks (men/6 weeks: *p* = .211, men/10 weeks: *p* = .844, women/6 weeks: *p* = .319, women/10 weeks: *p* = .319). For men of the control group, the size of waist circumference tended to increase from baseline at 10 weeks (*p* = .050). Table 4 also shows the stage of behaviour changes among this study's participants. In the intervention group, significant progress was confirmed in both the dietary habit and the exercise habit at 6 weeks (dietary habit: *p* = .025, exercise habit: *p* = .030). At 10 weeks, these stages of behaviour changes returned to the baseline in the intervention group.

**Table 4:** Changes of their waist circumference and TTM stage

	Intervention (*N* = 128)					Comparison of within groups	Intervention (*N* = 128)			Comparison of within groups	Control (*N* = 141)					Comparison of within groups	Comparison of between groups
	*N*	Baseline		6 weeks		*p* a	*N*	10 weeks		*p* a, c	*N*	Baseline		10 weeks		*p* a	*p* b, d
		Median	Mean ± *SD*	Median	Mean ± *SD*			Median	Mean ± *SD*			Median	Mean ± *SD*	Median	Mean ± *SD*		
Waist circumference (cm)																	
Men	105	80.0	81.71 ± 9.40	80.0	81.62 ± 9.16	.211	107	80.0	81.90 ± 9.36	.844	119	80.0	81.52 ± 9.33	80.5	82.00 ± 9.29	.050	.189
Women	12	70.5	71.57 ± 5.61	73.0	71.00 ± 6.77	.167	12	70.5	71.17 ± 6.74	.319	18	68.0	72.67 ± 9.82	68.0	71.78 ± 8.90	.567	.723
TTM stage																	
Dietary habit	121	2.0	2.01 ± 1.05	2.0	2.23 ± 1.10	.025	126	2.0	2.01 ± 0.92	.493	138	2.0	2.13 ± 1.08	2.0	2.01 ± 1.02	.179	.042
Exercise habit	120	2.0	2.05 ± 0.99	2.0	2.28 ± 1.12	.030	126	2.0	2.03 ± 0.95	.827	137	2.0	2.06 ± 1.03	2.0	2.15 ± 1.01	.330	.982
Smoking habit	77	1.0	2.12 ± 1.54	2.0	2.09 ± 1.34	.701	81	2.0	2.11 ± 1.38	.574	73	1.0	2.15 ± 1.52	1.0	2.00 ± 1.45	.586	.269

Abbreviation: TTM stage, transtheoretical model stage.

a Wilcoxon signed‐rank test.

b Mann–Whitney *U* test.

c Compare the data on baseline with the data on postintervention (10 weeks).

d Comparison of between groups on 10 weeks.

## 4 DISCUSSION

### 4.1 Changes in knowledge or cognition related to diabetes prevention and health management

A previous study had found that lack of experience related to disease brought about lack of interest in health in young adults and hindered motivation to change poor health‐related behaviours (Ozaki, Watai, & Miyagawa, 2017). Consequently, our intervention focused on educating young adults about diabetes prevention and health management and our study results suggested that such education could increase the immediacy of diabetes as a possible risk in that population. In early stages of behavioural change, the cognitive change is considered to be the most important factor (Pender, 2014). Table 4 shows that most of this study's participants assumed that their stage of behaviour change was the early stage. Furthermore, in the early stage of behaviour change, knowledge acquisition that promotes realization of unhealthy behaviours and the obstacles caused by them stimulates behaviour changes (Hata & Doi, 2009). Therefore, enhancing health cognition is a promising way to promote positive behaviour changes in young adults.

### 4.2 The PHCS scores

The PHCS measures how much an individual feels capable of modifying health‐related lifestyle and behaviour. In our study population, education did not increase this score, in fact, the score was decreased on average by a very small amount. The reason for this is unclear. The PHCS scores of this study's participants were lower than that of another study whose individuals' educational background and occupation were similar to ours (Togari, Yamazaki, Koide, & Miyata, 2006a). The PHCS score is known to be related to favourable healthy practices (Togari et al., 2006a, 2006b). Since our intervention group only showed improvement in two out of nine dietary behaviour items and no improvement in amount of daily exercise, the slightly reduced PHCS score after education may reflect recognition of diabetes risk paired with recognition that it is difficult to act on that information.

### 4.3 Dietary behaviours and the stage of behaviour change

In comparison to the controls, the intervention group showed significantly improvement in their dietary behaviour in regard to two items: taking more than 20 min per meal and consuming a well‐balanced meal with a staple, main dish and side dish. Spending less time eating has been identified as a factor greatly related to weight gain and the onset of metabolic syndrome for young adults (Hori, 2013; Soga et al., 2013). Thus, it is possible that our education programme might improve participants' health in future by encouraging the participants to spend more time on taking a meal. Furthermore, a previous study had pointed out that the following obstructive factors affected the health‐related behaviours of young obese males: “there is less recognition about the importance of the well‐balanced diet,” “it is difficult to have a healthy diet by reason of time required, labour and costs” (Ozaki et al., 2017). In contrast, our study interventions improved our participants' behaviour related to having well‐balanced meals with a staple, main dish and side dish. Therefore, this study may prevent obesity in future. Our educational programme provided photographs and cooking methods that reduce time and cost and may have been responsible for producing these positive effects. In addition, the intervention group showed improvement in consumption of the necessary amount of vegetables. A previous study found that the habitual practice of consuming vegetables and fruit daily was one of the predictors of completion of a primary healthcare diabetes prevention programme for individuals with high diabetes risk (Gilis‐Januszewska et al., 2018). We believe that to increase vegetable intake from the time one is young and healthy, the prevention programme should be modified to have content that increases motivation and is easier to put into practice.

Even though the stages of behaviour change about dietary habit significantly improved more in the intervention group at 6 weeks, it returned to the baseline at 10 weeks. Giving information and knowledge about favourable healthy practices is difficult to drive behaviour changes (Kelly & Barker, 2016; Tominaga et al., 2010). Therefore, we provide our participants information considering their interests. Our programme is possible to be a trigger of starting the behaviour change at 6 weeks. It is necessary to elaborate strategies for driving behaviour changes consistently.

### 4.4 The amount of physical activity and the stage of behaviour change

The amount of physical activity did not change with education. Even patients with a diagnosis of diabetes have trouble learning to exercise regularly despite specific rewards such as control of blood sugar and prevention of complications (Morrato, Hill, Wyatt, Ghushchyan, & Sullivan, 2007; Palakodeti, Uratsu, Schmittdiel, & Grant, 2015; Senba, Sato, Koga, & Fujita, 2009), so it is probably even harder for healthy young adults to prioritize exercise over jobs or time with family. Some factors that have been found to inhibit exercise are difficulty of securing free time and the desire to spend free time and money on more pleasurable activities (Tanbo & Inagaki, 2009). Moreover, a previous study supported diabetics’ behavioural changes using telephone follow‐ups and was also unable to improve exercise habits (Sakane et al., 2015). Studies that have been successful in bringing about continuous behavioural change with respect to exercise have involved group‐based exercise (Santanasto et al., 2017), a way to manage exercise records (Aguiar et al., 2017) and an interactive web‐based programme tailored to each individual (Jahangiry, Montazeri, Najafi, Yaseri, & Farhangi, 2017). Modifying our programme to include more intensive and interactive content and modifying the environment where our participants exercise may be necessary to improve our outcomes.

### 4.5 Smoking

About smoking, the smoking rate of both groups was about 50%. The national male smoking rate is about 40% in Japan (The Ministry of Health, Labor, & Welfare, 2016b). Our programme contained information about the relation between smoking and diabetes risk. A previous study found that recognition that smoking behaviour is destructive to health could be an influencing factor for achieving smoking cessation (Arai et al., 2016). However, young adults usually have not yet experienced the detrimental effects of smoking and they do not associate health with smoking cessation. But it is also known that the longer someone smokes, the harder it is for him or her to stop smoking (Arai et al., 2016). Accordingly, an education about the importance of smoking cessation should be provided early in life.

### 4.6 Waist circumference

The average waist circumference of our study participants was similar to the national average, and most of these study participants did not require weight loss. That may be one reason we observed no weight loss in the intervention group after our programme.

### 4.7 Strengths and Limitations

There are few studies that focus on educating healthy young adults about the importance of and methods for preventing diabetes, in accordance with their characteristics and lifestyles. Generally, it is difficult for getting young adults to become study participants, because they are usually busy after employment. But this study was able to recruit many such participants and is a strength. Unfortunately, previous programmes though effective (Knowler et al., 2002; Sakane et al., 2015; Tuomilehto et al., 2001) are very expensive because they require face to face guidance or frequent telephone‐delivered support by healthcare professionals (Hollenbeak, Weinstock, Cibula, Delahanty, & Trief, 2016). Henceforth, we should develop education methods that target healthy young adults to reduce costs. On the other hand, this study had mainly two limitations. First, the participants of this study were not collected from a broad spectrum of young adults but was limited to employees of automobile sales companies and the percentage of males was high. Many of the participants were mechanics, rather than desk workers, which may also limit generalizability of the results. In future, we would like to study young adults in a broad range of business types. Second, ours was not a randomized controlled trial and this study period was also short (10 weeks).

## 5 CONCLUSION

We found that educational intervention can raise awareness of diabetes as a common risk and enhance knowledge about its prevention in healthy young adults. Because Japanese people in general have a genetic susceptibility to T2DM, such interventions are highly desirable.

This education programme also showed that individuals in early adulthood are interested in improving their dietary habits and eating behaviours. We suggest developing educational programmes for young adults that suggest easy ways to alter their eating habits and that are feasible despite their busy lifestyle.

## CONFLICT OF INTEREST

The authors declare that they have no competing interests.

## AUTHOR CONTRIBUTIONS

HN & KM: Designed the intervention devices and questionnaires, analysed data. HN: Drafted the manuscript. All authors distributed brochures and films, read and approved the final manuscript.

## ETHICAL APPROVAL

Participants were informed about this study in writing before the start of this study. Consent for participation was assumed by return of questionnaires. We received approval from the Ethics Review Board of our university as an ethical consideration. This trial has been registered with UMIN‐CTR crinical trial (UMIN000023749).

## Data Availability

The datasets generated during and/or analysed during the current study are not publicly available due to stipulations about secondary use at the time the study was approved.
